# Comparison of early diabetic retinopathy staging in asymptomatic patients between autonomous AI-based screening and human-graded ultra-widefield colour fundus images

**DOI:** 10.1038/s41433-021-01912-4

**Published:** 2022-02-07

**Authors:** Aleksandra Sedova, Dorottya Hajdu, Felix Datlinger, Irene Steiner, Martina Neschi, Julia Aschauer, Bianca S. Gerendas, Ursula Schmidt-Erfurth, Andreas Pollreisz

**Affiliations:** 1 Department of Ophthalmology and Optometry, Medical University of Vienna, Vienna, Austria; 2 Center for Medical Statistics, Informatics and Intelligent Systems, Section for Medical Statistics, Medical University of Vienna, Vienna, Austria

**Keywords:** Retinal diseases, Medical imaging

## Abstract

**Introduction:**

Comparison of diabetic retinopathy (DR) severity between autonomous Artificial Intelligence (AI)-based outputs from an FDA-approved screening system and human retina specialists’ gradings from ultra-widefield (UWF) colour images.

**Methods:**

Asymptomatic diabetics without a previous diagnosis of DR were included in this prospective observational pilot study. Patients were imaged with autonomous AI (IDx-DR, Digital Diagnostics). For each eye, two 45° colour fundus images were analysed by a secure server-based AI algorithm. UWF colour fundus imaging was performed using Optomap (Daytona, Optos). The International Clinical DR severity score was assessed both on a 7-field area projection (7F-mask) according to the early treatment diabetic retinopathy study (ETDRS) and on the total gradable area (UWF full-field) up to the far periphery on UWF images.

**Results:**

Of 54 patients included (*n* = 107 eyes), 32 were type 2 diabetics (11 females). Mean BCVA was 0.99 ± 0.25. Autonomous AI diagnosed 16 patients as negative, 28 for moderate DR and 10 for having a vision-threatening disease (severe DR, proliferative DR, diabetic macular oedema). Based on the 7F-mask grading with the eye with the worse grading defining the DR stage 23 patients were negative for DR, 11 showed mild, 19 moderate and 1 severe DR. When UWF full-field was analysed, 20 patients were negative for DR, while the number of mild, moderate and severe DR patients were 12, 21, and 1, respectively.

**Conclusions:**

The autonomous AI-based DR examination demonstrates sufficient accuracy in diagnosing asymptomatic non-proliferative diabetic patients with referable DR even compared to UWF imaging evaluated by human experts offering a suitable method for DR screening.

## Abstract 2

**Learning Objectives:**

Upon completion of this activity, participants will:

1. Compare diabetic retinopathy (DR) severity scores of ophthalmologically asymptomatic people with diabetes between outputs from an artificial intelligence (AI)-based system and human-graded ultra-widefield (UWF) color fundus imaging, according to a clinical study.
2. Compare manual 7F-mask gradings vs UWF full-field gradings and describe the correlation with patient characteristics, according to a clinical study.
3. Describe clinical implications of the comparison between the DR severity scores of ophthalmologically asymptomatic people with diabetes outputs using outputs from an AI-based system and human-graded UWF color fundus imaging, according to a clinical study.

**Accreditation Statements:**

In support of improving patient care, this activity has been planned and implemented by Medscape, LLC and Springer Nature. Medscape, LLC is jointly accredited by the Accreditation Council for Continuing Medical Education (ACCME), the Accreditation Council for Pharmacy Education (ACPE), and the American Nurses Credentialing Center (ANCC), to provide continuing education for the healthcare team.

Medscape, LLC designates this Journal-based CME activity for a maximum of 1.0 *AMA PRA Category 1 Credit(s)™*. Physicians should claim only the credit commensurate with the extent of their participation in the activity.

Successful completion of this CME activity, which includes participation in the evaluation component, enables the participant to earn up to 1.0 MOC points in the American Board of Internal Medicine’s (ABIM) Maintenance of Certification (MOC) program. Participants will earn MOC points equivalent to the amount of CME credits claimed for the activity. It is the CME activity provider’s responsibility to submit participant completion information to ACCME for the purpose of granting ABIM MOC credit.

All other clinicians completing this activity will be issued a certificate of participation. To participate in this journal CME activity: (1) review the learning objectives and author disclosures; (2) study the education content; (3) take the post-test with a 75% minimum passing score and complete the evaluation at www.medscape.org/journal/eye; (4) view/print certificate.

**Credit hours:**

1.0

**Release date:**

**Expiration date:**

**Post-test link:** https://www.medscape.org/eye/posttest964708

**Authors/Editors disclosure information:**

S.S. has disclosed the following relevant financial relationships: Served as consultant or advisor for Allergan, Inc.; Bayer HealthCare Pharmaceuticals; Boehringer Ingelheim Pharmaceuticals, Inc.; Heidelberg Pharma GmbH; Novartis Pharmaceuticals Corporation; Optos; Roche; Served as a speaker or a member of a speakers bureau for Allergan, Inc.; Bayer HealthCare Pharmaceuticals; Boehringer Ingelheim Pharmaceuticals, Inc.; Novartis Pharmaceuticals Corporation; Optos; Roche; Received research funding from Bayer HealthCare Pharmaceuticals; Boehringer Ingelheim Pharmaceuticals, Inc.; Novartis Pharmaceuticals Corporation; Optos; Is employed by or has an executive role as Data Monitoring Chair for Phase 2 study sponsored by Bayer HealthCare Pharmaceuticals; Scientific Committee Member of EyeBio Steering Committee for FOCUS sponsored by Novo Nordisk. Other: Trustee member for Macular Society Scientific/Research Advisory Committee Member for Sight UK, Retina UK, Macular Society.

**Journal CME author disclosure information:**

Laurie Barclay has disclosed no relevant financial relationships.

## Introduction

Diabetic retinopathy (DR) is a vision-threatening disease affecting approximately one-third of individuals diagnosed with diabetes mellitus [1]. It has been predicted that by the year 2030 there will be 439 million adults affected worldwide, rising to an estimated 629 million by 2045 [2, 3]. The number of patients with vision-threatening DR is expected to increase dramatically over the next years [4]. Scientific and clinical evidence proved that early diagnosis and well-timed treatment are crucial in preventing visual loss in these patients [5].

Over the last decades, advances in machine learning and deep learning have made it possible to automatically identify various ophthalmological diseases from colour fundus images such as DR, age-related macular degeneration, or glaucoma [6–9].

Multiple automated algorithms for DR detection from retinal colour photographs have been developed [7, 10–12]. IDx-DR was the first autonomous artificial intelligence (AI)-based diagnostic system approved by the U.S. Food and Drug Administration (FDA). It consists of a robotic fundus camera and two types of algorithms, namely for image quality assessment as well as immediate diagnosis of the DR stage in case of sufficient image quality from four colour fundus images. IDx-DR provides one output per patient including both eyes. In a preregistered trial, IDx-DR was validated against the ETDRS protocol prognostic standard, and showed 87.2% sensitivity and 90.7% specificity for identifying ETDRS 35 and above, or any form of macular oedema, which includes moderate and vision-threatening DR that require consultation of an ophthalmologist [13, 14].

To date there are several different classification systems for DR. The Airlie House Classification, which was modified for the Early Treatment Diabetic Retinopathy Study (ETDRS), remains the gold standard for diagnosis of DR in a research setting as it correlates with the risk of DR progression [15, 16]. Stereoscopic images with a field of 30° of the standard 7-fields are evaluated and graded in 13 severity levels, ranging from 10 (no diabetic retinopathy) to 85 (e.g. severe retinopathy with retinal detachment at macula) [16]. In order to simplify DR classification for clinical use, the International ﻿Clinical Disease (ICDR) Severity Scale was introduced according to the findings of ETDRS and ﻿the Wisconsin Epidemiologic Study of Diabetic Retinopathy (WESDR). Five stages of DR were described as following—‘no apparent retinopathy,’ ’mild non-proliferative retinopathy (NPDR),’ ‘moderate NPDR,’ ‘severe NPDR,’ ‘proliferative diabetic retinopathy (PDR).’ Additionally, clinically significant and centre-involved diabetic macular oedema (DMO) can occur in any stage of DR [17].

With modern imaging modalities such as widefield (WF) imaging and ultra-widefield (UWF) imaging of the retina, it is now possible to obtain valuable information from peripheral retinal areas that could otherwise be missed with conventional imaging [18]. It has been demonstrated that diabetic retinal lesions are present in areas outside the standardised 7 ETDRS fields in about 40% of diabetic eyes, resulting in more severe DR levels in 10% of eyes [19, 20]. However, the prognostic impact of these peripheral lesions, if any, is subject to study.

WF images are defined to depict the retina in all 4 quadrants up to and including the region of the vortex vein ampullae, while UWF images extend the field of view beyond their anterior edge [21]. Current laser-based retinal imaging systems allow the capture of WF or UWF images either by image montages or a single-shot, visualising a field of view of up to 200°, which corresponds to about 82% of the total retinal area [21, 22]. A new DR staging system is under development, and UWF and other new modalities are being considered for being part of it [23].

In this study, we aimed to compare DR severity scores of ophthalmologically asymptomatic people with diabetes between outputs from an autonomous AI-based system (IDx-DR, Digital Diagnostics) and human-graded UWF colour images including the overlay of an ETDRS 7-field area.

## Methods

### Subjects

The present prospective observational pilot study was performed in adherence to the Declaration of Helsinki including current revisions and the Good Clinical Practice guidelines. Informed written consent had been acquired prior to the inclusion in this study and the approval of the Ethics Committee of the Medical University of Vienna (MUV) was received. Individuals diagnosed with diabetes mellitus (type 1 and 2) without any subjective visual complaints, no known previous diagnosis of DR, no confounding eye diseases, and no known laser photocoagulation prior to the study, were recruited at the Department of Ophthalmology (MUV).

### Image acquisition and autonomous diagnosis with the IDx-DR system

**Fig. 1:**
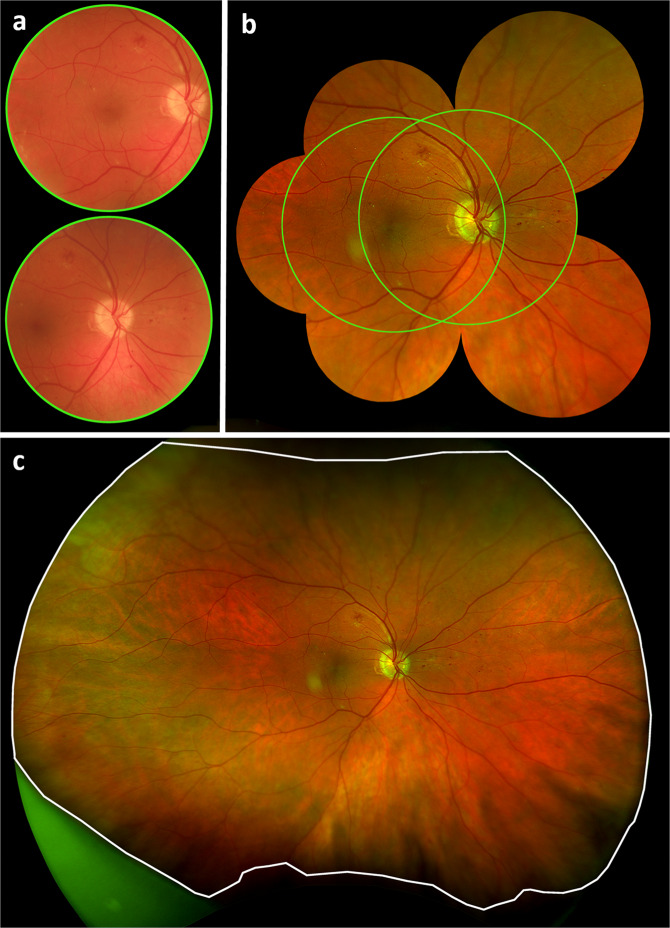
Comparison of different field of views obtained by Topcon and Optomap fundus imaging systems. **a** Two 45° fundus images (Topcon TRC-NW400 non-mydriatic fundus camera) of the right eye of a male patient (60 years) with moderate DR centred on the macula and on the optic disc. **b** UWF image (Optos, Daytona) of the same patient with masked periphery and only 7F-mask area and the area of the autonomous AI analysis (green circles) are visible. **c** UWF image of the same patient with full-field area outlined in white.

Patients were diagnosed with IDx-DR V2.2 at MUV by a trained operator. Of each eye, two 45° colour fundus images from the centre of the macula and the optic disc were captured using the Topcon TRC-NW400 non-mydriatic fundus camera (Topcon Medical Systems, Inc.), and automatically checked by the Image Quality Assessment tool (Fig. 1A). The Diagnostic Algorithm evaluated the presence of DR with three possible outputs: no/mild DR (labelled as negative), moderate, and vision-threatening DR (including severe DR, proliferative DR and DMO in any DR stage). For all four images of the same patient one output is provided by IDx-DR, which constitutes the higher DR stage of both eyes.

### Subject examination

Detailed ophthalmological examination was performed after autonomous AI imaging at the Department of Ophthalmology and Optometry at the MUV including best-corrected visual acuity (BCVA) with Snellen charts, UWF colour fundus imaging with Optomap (Daytona, Optos), spectral-domain optical coherence tomography (SD-OCT) imaging (Heidelberg Engineering, Germany) and a fundus examination with a slit-lamp microscopy to exclude any other retinal abnormalities than DR. Subjects presenting with centre-involved DMO as defined by DRCR Retina Network as retinal thickness in the ETDRS central subfield of 250 μm or greater on OCT, as well as non-centre-involved DMO in the inner and outer ETDRS rings were excluded [24].

Age, gender, history of stroke, myocardial infarction, the presence of arterial hypertension, hyperlipidaemia, current haemoglobin A1C (HbA1c) levels, insulin intake, and concomitant glaucoma were recorded from a patient questionnaire.

### Image acquisition and analysis with Optomap

UWF colour fundus images were captured with Optomap (Daytona, Optos). A 7-field ETDRS area (7F-mask) was overlaid in the Optos Advance software (version 4.231.94248) (Fig. 1B). The 7F-mask areas were graded by two retina specialists (AS and DH) according to a protocol similar to the ETDRS Scale of Diabetic Retinopathy Severity Scale to assess DR levels using a 21-inch display with a 1080p resolution. These levels were matched with the corresponding ICDR Severity Scale, which is the basis for the autonomous AI outputs. The area outside of the ETDRS 7-fields was also evaluated and ICDR levels were assessed in the total gradable area (UWF full-field) extending to the far periphery (Fig. 1C).

ICDR grading is defined as follows. In case only microaneurysms are present, the disease severity level is mild DR. If more than only microaneurysms, including a single haemorrhage, can be observed, the level is moderate DR. Severe DR is diagnosed if any of the following conditions are true: extensive intraretinal haemorrhages in each of 4 quadrants, definite venous beading in more than 2 quadrants, prominent intraretinal microvascular anomalies (IRMA) in one or more quadrant and no signs of proliferative DR.

Manual grading of the DR stage on colour fundus images obtained by the Optomap system was either indicated per eye or to be in line with the approach used by the AI system per patient.

### Statistical analysis

Statistical calculations were performed using R 3.6.2. Quantitative variables are summarised mean±standard deviation (SD). For qualitative variables absolute and relative frequency (%) are reported. Prevalence of negative/mild and moderate/severe DR were determined by autonomous AI, which has been previously validated against full ETDRS and DRCR prognostic standards as well as by UWF grading [13].

Agreements between the grading of 7F-mask and autonomous AI outputs, between UWF full-field and autonomous AI outputs, respectively, were analysed with κ (R-package vcd, R-function Kappa). Estimates for sensitivity, specificity, positive and negative predictive value with 95% Clopper-Pearson confidence limits were reported, whereby 7F-mask or UWF full-field was taken as a gold standard.

The gradings were dichotomised in negative/mild and moderate/severe. If the measurements differed between eyes, the worse eye of each patient was determined. Hence, only one eye per patient was considered in the statistical analyses.

The agreement between the gradings (taken as an ordinal variable) of 7F-mask and UWF full-field was analysed by contingency table and weighted κ, including both eyes of each patient. To determine the 95% confidence limit of κ, the confidence limits were calculated for the left and right eye separately. The lower 95% confidence limit of the κ was then determined as the minimum of the lower 95% limit of left and right eye and the upper limit as the maximum of the 95% upper limit of left and right eye, respectively.

The correlation between autonomous AI outputs and 7F-mask gradings, respectively, and BCVA, diabetes duration and type, gender, HbA1c, history of myocardial infarction and stroke, arterial hypertension, hyperlipidaemia, glaucoma, and insulin intake, respectively, were analysed with Spearman’s correlation with 95% confidence limits and two-sided *p*-values (H0: rho = 0).

Autonomous AI output and 7F-mask gradings were taken as ordinal variables. One eye per patient (the eye with the higher 7F-mask grading) was included in the analyses. If 7F-mask gradings were equal for both eyes, the mean BCVA of the patient was analysed. The significance level was set to 0.05. The interpretation of the *p*-values is descriptive.

## Results

This study included 107 eyes of 54 patients (33 male, 21 female) with a mean age of 55 ± 15.5 years (range: 19–80 years). Out of these patients, 32 had type 2 diabetes (11 females) with a mean HbA1c of 7.5 ± 1.9% and 20 had type 1 diabetes (8 females) with a mean HbA1c of 7.9 ± 1.6%, in two patients the diabetes type was unknown. Table 1 summarises patient characteristics.

**Table 1:** Patient characteristics.

No. of patients/eyes	54/107
Gender, number (%)	
Male	33 (61.1)
Female	21 (38.9)
Diabetes type, number of patients (%)	
1	20 (37.0)
2	32 (59.3)
No records	2 (3.7)
Hb1A1C level (%), mean ± SD	7.6 ± 1.7
BCVA, mean ± SD	0.99 ± 0.25
Arterial hypertension, no. (%)	21 (38.2)
No records	1 (1.8)
Hyperlipidemia, no. (%)	19 (35.2)
No records	1 (1.9)
History of stroke, no. (%)	2 (3.6)
No records	4 (7.3)
History of myocardial infarction, no. (%)	2 (3.6)
No records	4 (7.3)
Glaucoma, no. (%)	3 (5.5)
No records	1 (1.8)
Insulin therapy (%)	31 (56.4)
No records	1 (1.8)

The autonomous AI-based system diagnosed 16 patients (29.6%) with no or mild DR, 28 patients (51.9%) with moderate DR, and 10 patients (18.5%) with vision-threatening DR. One UWF image of 1 eye was excluded due to insufficient image quality. 7F-mask grading diagnosed 23 patients (42.6%) with no DR, 11 (20.4%) with mild, 19 (35.2%) with moderate, and 1 (1.9%) with severe DR. UWF full-field (entire imageable retina) diagnosed 20 patients (37.0%) presented with no DR, and 12 (22.2%) with mild, 21 (38.9%) with moderate and 1 (1.9%) with severe DR.

### Comparison of automated outputs vs. manual gradings per patient

Grouping the manually graded DR readouts according to the autonomous AI outputs of no/mild DR, moderate DR, and vision-threatening DR, which corresponds to severe DR, and comparing these results to the autonomous AI output revealed the following. In 66.6% of cases, autonomous AI and 7F-mask gradings matched (Table 2, κ [95% CI]: 0.4 [0.21; 0.58]), while autonomous AI and UWF full-field corresponded in 66.7% of cases (Table 3, κ [95% CI]: 0.38 [0.18; 0.58]). In one case autonomous AI output matched 7F-mask grading as no/mild DR, whereas UWF full-field grading showed moderate DR, as a result of haemorrhages present in the peripheral fields, outside the 2 fields available to the AI, and the 7-fields available to 7F-mask grading. Correspondingly, the autonomous AI sensitivity/specificity against the 7F-mask grading was 100% (95% CI: 83–100)/47% (95% CI: 30-65), and against UWF full-field grading 95% (95% CI: 77–100)/47% (95% CI: 29–65). The positive/negative predictive value of AI against the 7F-mask grading was 53% (95% CI: 36–69)/100% (95% CI: 79–100), and against UWF full-field grading 55% (95% CI: 38–71)/94% (95% CI: 70–100).

**Table 2:** Comparison of autonomous AI outputs with 7F-mask area grading on UWF images with DR stage of the patient defined by the eye with the worse DR stage.

	Autonomous AI output, no. patients (%)	
	No DR/mild DR	Moderate/vision-threatening (severe DR)
7F-mask area grading on UWF images, no. patients (%)		
No DR/mild DR	16 (29.6%)	18 (33.3%)
Moderate DR/severe DR	0	20 (37.0%)

**Table 3:** Comparison of autonomous AI output with UWF full-field grading with DR stage of the patient defined by the eye with the worse DR stage.

	Autonomous AI output, no. patients (%)	
	No DR/mild DR	Moderate/vision-threatening (severe DR)
UWF full-field grading, no. patients (%)		
No DR/mild DR	15 (27.8%)	17 (31.5%)
Moderate DR/severe DR	1 (1.9%)	21 (38.9%)

### Comparison of right and left eyes

The per eye analysis of 7F-mask gradings revealed that 55 eyes (50.9%) were graded as negative for DR, 22 (20.4%) showed mild, 29 (26.9%) moderate, and 1 (0.9%) severe DR. When UWF full-field was analysed, 48 eyes (44.4%) were negative for DR, 24 eyes (22.2%) showed mild, 34 eyes (31.5%) moderate, and 1 eye (0.9%) severe DR.

Based on the 7F-mask grading of colour fundus images, there were 17 patients (31.5%) with different DR stages between left and right eyes. When comparing the worse DR grade of both eyes to the AI output in the categories no/mild DR, moderate DR, or vision-threatening DR (severe DR), there was an agreement in 12 out of the 17 patients (70.6%, κ [95% CI]: 0.21 [−0.15; 0.56]) (Table 4A). In other cases, autonomous AI differed from 7F-mask human grading by showing higher DR severity.

**Table 4:** A. Patients with different DR stages between eyes diagnosed with 7F-mask area grading on UWF images. B. Patients with different DR stages between eyes were diagnosed with UWF full-field grading.

(A) Patients, no.	7F-mask area grading on UWF images		Autonomous AI output
	Eye A	Eye B	
4	Moderate DR	Mild DR	Moderate DR
3	No DR	Moderate DR	Moderate DR
1	Moderate DR	Severe DR	Vision-threatening DR
1	No DR	Mild DR	negative
5	No DR	Mild DR	Moderate DR
3	Moderate DR	Mild DR	Vision-threatening DR

(B) Patients, no.	UWF images grading		IDx-DR output
	Eye A	Eye B	
4	Moderate DR	Mild DR	Moderate DR
4	No DR	Moderate DR	Moderate DR
1	Moderate DR	Severe DR	Vision-threatening DR
1	No DR	Mild DR	negative
3	No DR	Mild DR	Moderate DR
1	Moderate DR	Mild DR	Vision-threatening DR

Based on UWF full-field gradings, there were 14 patients (25.9%) with different DR stages between left and right eyes. Again, when comparing the worse DR stage to the autonomous AI output in the categories no/mild DR, moderate DR, or vision-threatening DR (severe DR), an agreement between autonomous AI outputs and manual gradings could be found in 11 patients (78.6%, κ [95% CI]: 0.32 [-0.18; 0.82]) (Table 4B). In other cases, autonomous AI differed from UWF human grading by showing a higher DR severity.

### Comparison of manual 7F-mask gradings vs. ultra-widefield full-field gradings

Comparison of 7F-mask grading with classification based on ETDRS criteria on UWF full-field images showed very good agreement (weighted κ [95% CI]: 0.88 [0.76; 0.99], *n* = 107 eyes of 54 patients). In total, 12 eyes (11.1%) were classified with a more severe DR grade when UWF full-field was included in the analysis. The estimate of the severity of DR increased by 1 grade, meaning from no to mild DR in 7 eyes (6.4%) as a result of microaneurysms present in the peripheral fields on UWF grading, outside the 7 ETDRS fields and from mild to moderate DR in 5 eyes (4.6%) due to the presence of peripheral haemorrhages. The 7F-mask grading sensitivity/specificity against UWF was 91% (95% CI: 71–99)/100% (95% CI: 89–100), and the positive predictive value/negative predictive value was 100% (95% CI: 83–100)/94% (95% CI: 80–99).

### Correlation with patient characteristics

We found weak correlations between BCVA, diabetes duration and type, gender, history of myocardial infarction and stroke, insulin intake, arterial hypertension, hyperlipidaemia, glaucoma, and autonomous AI grading (Spearman’s correlation ranging from −0.15 to 0.27) and 7F-mask grading (Spearman’s correlation ranging from −0.22 to 0.20) and moderate positive correlations between HbA1c and autonomous AI (rs [95% CI]: 0.41 [0.15; 0.62], *p* = 0.003, *n* = 49) and between HbA1c and 7F-mask grading (rs [95% CI]: 0.51 [0.27; 0.70], *p* = 0.0002, *n* = 49).

## Discussion

We compared DR stages from ophthalmologically asymptomatic diabetes patients by outputs from an autonomous AI-based system (IDx-DR) and human grading of colour fundus images up to the far periphery of the retina. In 66.6% (*n* = 36) of patients, the gradings of autonomous AI matched those of 7F-mask grading and no case showed a more severe disease stage in the 7F-mask grading. When UWF full-field was analysed, only 1 out of 54 patients demonstrated a more severe DR stage, compared to the output of autonomous AI (moderate manual grading and no/mild autonomous AI output). In this case, one haemorrhage could be seen in the periphery.

Early detection and treatment of referable DR is of utmost importance to prevent vision loss in diabetic patients. The introduction of autonomous screening systems based on deep learning methods enables examining large numbers of patients even in remote areas lacking easy access to ophthalmologists [25].

Readouts of the IDx-DRs are based on the evaluation of two 45° fundus images per eye, of which one is macula-centred and the other fovea-centred with minimal overlap between the 2 images with one output per patient. These two images represent less than half of the area depicted by the standard 7-field ETDRS area. Furthermore, training of staff for handling the fundus camera requires a minimal expenditure of time with no more than 4 h instruction needed for the personnel on-site [13]. IDx-DR was applied in a real-life setting in a Dutch population of 1410 patients with a reported sensitivity of 68% and specificity of 86% compared to the ICDR grading [26]. Abramoff et al. showed in a preregistered clinical trial with 900 diabetic patients that the overall sensitivity for detecting more than mild DR by IDx-DR was 87.2% with a specificity of 90.7% compared to images corresponding to a 7-field ETDRS area as well as macular OCT analysed by a professional reading centre [13]. This study was the basis for IDx-DR de novo FDA authorisation as the first autonomous-AI diagnostic system. A recent study conducted by Shah et al. in a Spanish population of 2680 subjects revealed 100% sensitivity and 82% specificity for IDx-DR for detecting referable DR and 100% sensitivity and 95% specificity for detecting vision-threatening DR compared to manual gradings [27].

Because IDx-DR is validated for identifying more than mild DR in the U.S., we were able to compare human gradings of no/mild DR or moderate/severe DR with the equivalent autonomous AI gradings of no/mild DR or moderate/vision-threatening DR. In 66.6% (*n* = 36) of cases, the autonomous AI outputs matched 7F-mask gradings. The remaining 33.4% (*n* = 18) of the patients were graded as having either moderate or vision-threatening DR with autonomous AI, while they were diagnosed with either no or mild DR by retina specialists. There are several potential confounding factors contributing to this difference in diagnosis: media opacifications, vitreous floaters or other artifacts that could be projected on the image of the retina, potentially obscuring it. Because vision-threatening DR includes severe DR, proliferative DR or macula oedema in any DR stage, it is possible that any presence of intraretinal cysts or hard exudates affects autonomous AI grading, leading to vision-threatening output. Patients presenting with centre and/or non-centre-involved DMO on SD-OCT (definition: methods) were excluded.

For grading, IDx-DR makes a decision based on the higher DR stage of both eyes seen on the four images of the patient. For example, no/mild DR in the left eye and moderate DR in the right eye is graded by autonomous AI as moderate DR, which would imply a consultation with an ophthalmologist.

A further study that analysed retinal images of diabetic patients (5084 cases) from eyePACS database with AI software (Eye Art) showed 90% sensitivity and 63% specificity diagnosing referable DR (moderate DR or worse or presence of DMO or ungradable image, respectively) compared to human graders [28]. Rajalakshmi et al. investigated fundus images of 296 diabetic patients acquired with a smartphone-based device, which were graded with AI screening software (Eye Art) and compared to ophthalmologist´s gradings and reported ﻿95.8% sensitivity and 80.2% specificity for detecting any DR [29]. Ting et al. evaluated 76370 retinal images by a deep learning system for identifying referable and vision-threatening DR and showed high sensitivity (90.5% for referable DR, 100% for vision-threatening DR) and specificity (91.6% for referable DR, 91.1% for vision-threatening DR) in their study research setting (no clinical recruitment) [6].

It has been demonstrated in previous studies that pathologic changes in DR occur in peripheral fields outside of the 7-field ETDRS area [30, 31]. Recently, advances in retinal imaging technologies have allowed peripheral retinal imaging to become routinely available [18, 32].

In a number of studies standard, ETDRS 7-field grading was compared with UWF colour fundus images for the diagnosis of DR [33–35]. It has been demonstrated that both imaging modalities can be used successfully for DR grading [34, 35]. However, DR changes outside ETDRS 7-fields result in more severe DR levels in 9 to 15% of the eyes examined. [20, 36–38] After assessing the DR severity of 206 eyes, Silva et al. showed a more severe DR level in 10% of eyes due to the presence of microaneurysms, haemorrhages, IRMA and new vessels elsewhere outside of the 7-field ETDRS area [20]. In another study evaluating 502 eyes with DR Silva et al. showed that 9% of eyes were classified with a more severe DR stage on UWF images compared to ETDRS 7-fields [37]. Comparison of a 7-field ETDRS with UWF grading by Aiello et al. revealed a DR level worsening in 11% of eyes [36]. Assessment of Optomap UWF images (*n* = 266) with a projected 7-field area by Price et al. showed 15% of eyes with a more severe DR stage [38]. These results correspond with our results of 11.1% of eyes showing a more severe DR level when UWF full-field was analysed manually compared to the manual grading of the 7F-mask area.

Silva et al. suggested that the presence and increasing number of DR lesions located mostly outside of 7-field ETDRS area positively correlate with DR worsening over 4 years [19].

There are several limitations to this study. First, the grading was performed using UWF colour fundus images and not stereoscopic images, which are considered to be the gold standard for DR diagnosis. This fact makes it nearly impossible to identify the presence of DMO. However, patients with DMO on OCT were excluded from this study. Potentially, the AI might have identified cases of DMO not identified on 7-field or UWF non-stereo. Second, the current sample size is limited to 107 eyes of 54 patients. Third, included patients were recruited from a tertiary referral centre, which makes our patient sample not representative of the general population and explains the high number of positive cases.

In conclusion, an FDA-authorised autonomous-AI diagnostic system demonstrated sufficient diagnostic accuracy for diagnosing early DR in asymptomatic non-proliferative diabetic patients compared to human expert gradings of the 7F-mask area on UWF colour images, making it suitable for DR screening and diagnosis in diabetes primary care settings or telemedicine programmes.

## Summary

### What was known before

- Diabetic lesions are present in areas outside the standardised 7 ETDRS fields, resulting in more severe DR levels in 10% of eyes.
- New diabetic retinopathy classification is under development and UWF imaging considered being a part of it.

### What this study adds

- First study comparing diabetic retinopathy grading between autonomous AI system and human-graded ultra-widefield colour fundus images.
- Even in comparison with UWF imaging the autonomous AI-based DR examination demonstrates sufficient accuracy in diagnosing asymptomatic non-proliferative diabetic patients with referable DR.

## Supplementary information

CME COMPONENTS FOR MEDSCAPE

## References

[1] Yau JWY, Rogers SL, Kawasaki R, Lamoureux EL, Kowalski JW, Bek T (2012). Global prevalence and major risk factors of diabetic retinopathy. Diabetes Care.

[2] Whiting DR, Guariguata L, Weil C, Shaw J (2011). IDF Diabetes Atlas: global estimates of the prevalence of diabetes for 2011 and 2030. Diabetes Res. Clin Pr.

[3] World Health Organization. (2016). Global report on diabetes. ISBN.

[4] Zheng Y, He M, Congdon N (2012). The worldwide epidemic of diabetic retinopathy. Indian J Ophthalmol.

[5] Liew G, Michaelides M, Bunce C (2014). A comparison of the causes of blindness certifications in England and Wales in working age adults (16-64 years), 1999-2000 with 2009-2010. BMJ Open.

[6] Ting DSW, Cheung CYL, Lim G, Tan GSW, Quang ND, Gan A (2017). Development and validation of a deep learning system for diabetic retinopathy and related eye diseases using retinal images from multiethnic populations with diabetes. JAMA.

[7] Abràmoff MD, Lou Y, Erginay A, Clarida W, Amelon R, Folk JC (2016). Improved automated detection of diabetic retinopathy on a publicly available dataset through integration of deep learning. Investig Ophthalmol Vis Sci.

[8] Burlina PM, Joshi N, Pacheco KD, Freund DE, Kong J, Bressler NM (2018). Use of deep learning for detailed severity characterization and estimation of 5-year risk among patients with age-related macular degeneration. JAMA Ophthalmol.

[9] Li F, Wang Z, Qu G, Song D, Yuan Y, Xu Y (2018). Automatic differentiation of Glaucoma visual field from non-glaucoma visual filed using deep convolutional neural network. BMC Med Imaging..

[10] Hipwell JH, Strachan F, Olson JA, McHardy KC, Sharp PF, Forrester JV (2000). Automated detection of microaneurysms in digital red-free photographs: a diabetic retinopathy screening tool. Diabet Med.

[11] Abramoff MD, Reinhardt JM, Russel SR, Folk JC, Mahajan VB, Niemeijer M (2010). Early detection of diabetic retinopathy. Ophthalmology..

[12] Sim DA, Keane PA, Tufail A, Egan CA, Aiello LP, Silva PS (2015). Automated retinal image analysis for diabetic retinopathy in telemedicine. Curr Diab Rep..

[13] Abràmoff MD, Lavin PT, Birch M, Shah N, Folk JC (2018). Pivotal trial of an autonomous AI-based diagnostic system for detection of diabetic retinopathy in primary care offices. npj Digit Med.

[14] Abramoff MD, Cunningham B, Patel B, Eydelman MB, Leng T. Foundational considerations for artificial intelligence utilizing ophthalmic images. Ophthalmology. 2021. 10.1016/j.ophtha.2021.08.023. 10.1016/j.ophtha.2021.08.023 PMC9175066 34478784

[15] Anon. Early Treatment Diabetic Retinopathy Study Research Group. (1991). Grading diabetic retinopathy from stereoscopic color fundus photographs—an extension of the modified airlie house classification: ETDRS Report Number 10. Ophthalmology.

[16] Anon. Early Treatment Diabetic Retinopathy Study Research Group. (1991). Fundus photographic risk factors for progression of diabetic retinopathy: ETDRS Report Number 12. Ophthalmology.

[17] Wilkinson CP, Ferris FL, Klein RE, Lee PP, Agardh CD, Davis M (2003). Proposed international clinical diabetic retinopathy and diabetic macular edema disease severity scales. Ophthalmology..

[18] Kim EL, Moshfeghi AA (2015). Wide-field imaging of retinal diseases. US Ophthalmic Rev.

[19] Silva PS, Cavallerano JD, Haddad NMN, Kwak H, Dyer KH, Omar AF (2015). Peripheral lesions identified on ultrawide field imaging predict increased risk of diabetic retinopathy progression over 4 years. Ophthalmology..

[20] Silva PS, Cavallerano JD, Sun JK, Soliman AZ, Aiello LM, Aiello LP (2013). Peripheral lesions identified by mydriatic ultrawide field imaging: distribution and potential impact on diabetic retinopathy severity. Ophthalmology..

[21] Choudhry N, Duker JS, Freund KB, Kiss S, Querques G, Rosen R (2019). Classification and guidelines for widefield imaging: recommendations from the International Widefield Imaging Study Group. Ophthalmol Retin.

[22] Patel M, Kiss S (2014). Ultra-wide-field fluorescein angiography in retinal disease. Curr Opin Ophthalmol..

[23] Sun JK, Aiello LP, Abràmoff MD, Antonetti DA, Dutta S, Pragnell M (2021). Updating the staging system for diabetic retinal disease. Ophthalmology..

[24] Mandelcorn E, Kertes PJ (2009). Three-year follow-up of a randomized trial comparing focal/grid photocoagulation and intravitreal triamcinolone for diabetic macular edema: commentary. Evid-Based Ophthalmol.

[25] Bellemo V, Lim ZW, Lim G, Nguyen QD, Xie Y, Yip MYT (2020). Artificial intelligence using deep learning to screen for referable and vision-threatening diabetic retinopathy in Africa: a clinical validation study. Lancet Digit Heal.

[26] van der Heijden AA, Abramoff MD, Verbraak F, van Hecke MV, Liem A, Nijpels G (2018). Validation of automated screening for referable diabetic retinopathy with the IDx-DR device in the Hoorn Diabetes Care System. Acta Ophthalmol.

[27] Shah A, Clarida W, Amelon R, Hernaez-Ortega MC, Navea A, Morales-Olivas J (2021). Validation of automated screening for referable diabetic retinopathy with an autonomous diagnostic artificial intelligence system in a Spanish Population. J Diabetes Sci Technol..

[28] Bhaskaranand M, Ramachandra C, Bhat S, Cuadros J, Nittala MG, Sadda S (2016). Automated diabetic retinopathy screening and monitoring using retinal fundus image analysis. J Diabetes Sci Technol..

[29] Rajalakshmi R, Subashini R, Mohan R, Viswanathan A. Automated diabetic retinopathy detection in smartphone-based fundus photography using artificial intelligence. Eye. 2018;32:1138–44. 10.1038/s41433-018-0064-9 PMC5997766 29520050

[30] Wessel MM, Aaker GD, Parlitsis G, Cho M, D’Amico DJ, Kiss S (2012). Ultra-wide-field angiography improves the detection and classification of diabetic retinopathy. Retina..

[31] Shimizu K, Kobayashi Y, Muraoka K (1981). Midperipheral fundus involvement in diabetic retinopathy. Ophthalmology..

[32] Ghasemi Falavarjani K, Wang K, Khadamy J, Sadda SR (2016). Ultra-wide-field imaging in diabetic retinopathy; an overview. J Curr Ophthalmol..

[33] Rasmussen ML, Broe R, Frydkjaer-Olsen U, Olsen BS, Mortensen HB, Peto T (2015). Comparison between Early Treatment Diabetic Retinopathy Study 7-field retinal photos and non-mydriatic, mydriatic and mydriatic steered widefield scanning laser ophthalmoscopy for assessment of diabetic retinopathy. J Diabetes Complications..

[34] Kernt M, Hadi I, Pinter F, Seidensticker F, Hirneiss C, Haritoglou C (2012). Assessment of diabetic retinopathy using nonmydriatic ultra-widefield scanning laser ophthalmoscopy (Optomap) compared with ETDRS 7-field stereo photography. Diabetes Care.

[35] Silva PS, Cavallerano JD, Sun JK, Noble J, Aiello LM, Aiello LP (2012). Nonmydriatic ultrawide field retinal imaging compared with dilated standard 7-field 35-mm photography and retinal specialist examination for evaluation of diabetic retinopathy. Am J Ophthalmol..

[36] Aiello LP, Odia I, Glassman AR, Melia M, Jampol LM, Bressler NM (2019). Comparison of early treatment diabetic retinopathy study standard 7-field imaging with ultrawide-field imaging for determining severity of diabetic retinopathy. JAMA Ophthalmol.

[37] Silva PS, Cavallerano JD, Tolls D, Omar A, Thakore K, Patel B (2014). Potential efficiency benefits of nonmydriatic ultrawide field retinal imaging in an ocular telehealth diabetic retinopathy program. Diabetes Care.

[38] Price LD, Au S, Chong NV (2015). Optomap ultrawide field imaging identifies additional retinal abnormalities in patients with diabetic retinopathy. Clin Ophthalmol.

