# Supplementary material for: Comparison of early diabetic retinopathy staging in asymptomatic patients between autonomous AI-based screening and human-graded ultra-widefield colour fundus images
Source: Eye (Lond). 2022 Feb 7;36(3):510–6. doi: 10.1038/s41433-021-01912-4 (PMC8873196; doi:10.1038/s41433-021-01912-4)
Supplement: Supplementary file 1 — CME COMPONENTS FOR MEDSCAPE [file 41433_2021_1912_MOESM1_ESM.docx]

**CME COMPONENTS**

**Journal:** Eye

**Issue:** January 2022

**Activity Title:** Comparison of Early Diabetic Retinopathy Staging in Asymptomatic Patients Between Autonomous AI-Based Screening and Human-Graded Ultra-Widefield Colour Fundus Images

**Post-Test Link:** <https://www.medscape.org/eye/posttest964708>

| Learning Objectives |
| --- |
| Upon completion of this activity, participants will:   1. Compare diabetic retinopathy (DR) severity scores of ophthalmologically asymptomatic people with diabetes between outputs from an artificial intelligence (AI)-based system and human-graded ultra-widefield (UWF) color fundus imaging, according to a clinical study 2. Compare manual 7F-mask gradings vs UWF full-field gradings and describe correlation with patient characteristics, according to a clinical study 3. Describe clinical implications of the comparison between DR severity scores of ophthalmologically asymptomatic people with diabetes outputs using outputs from an AI-based system and human-graded UWF color fundus imaging, according to a clinical study |

Credit Hours –1.0

Accreditation Statements

In support of improving patient care, this activity has been planned and implemented by Medscape, LLC and Springer Nature. Medscape, LLC is jointly accredited by the Accreditation Council for Continuing Medical Education (ACCME), the Accreditation Council for Pharmacy Education (ACPE), and the American Nurses Credentialing Center (ANCC), to provide continuing education for the healthcare team.

Medscape, LLC designates this Journal-based CME activity for a maximum of 1.0 *AMA PRA Category 1 Credit(s)™*. Physicians should claim only the credit commensurate with the extent of their participation in the activity.

Successful completion of this CME activity, which includes participation in the evaluation component, enables the participant to earn up to 1.0 MOC points in the American Board of Internal Medicine's (ABIM) Maintenance of Certification (MOC) program. Participants will earn MOC points equivalent to the amount of CME credits claimed for the activity. It is the CME activity provider's responsibility to submit participant completion information to ACCME for the purpose of granting ABIM MOC credit.

EDITOR

Sobha Sivaprasad, MD

Editor, *Eye*

Disclosure: Sobha Sivaprasad, MD, has disclosed the following relevant financial relationships:

Consultant or advisor for the following ineligible company(ies): Allergan, Inc.; Bayer HealthCare Pharmaceuticals; Boehringer Ingelheim Pharmaceuticals, Inc.; Heidelberg Pharma GmbH; Novartis Pharmaceuticals Corporation; Optos; Roche

Speaker or a member of a speakers bureau for the following ineligible company(ies): Allergan, Inc.; Bayer HealthCare Pharmaceuticals; Boehringer Ingelheim Pharmaceuticals, Inc.; Novartis Pharmaceuticals Corporation; Optos; Roche

Receive research funding from the following ineligible company(ies): Bayer HealthCare Pharmaceuticals; Boehringer Ingelheim Pharmaceuticals, Inc.; Novartis Pharmaceuticals Corporation; Optos

Employed by or have executive role with the following ineligible company(ies): Data Monitoring Chair for Phase 2 study sponsored by Bayer HealthCare Pharmaceuticals; Scientific Committee Member of EyeBio Steering Committee for FOCUS sponsored by Novo Nordisk

Other: Trustee member for Macular Society Scientific/ Research Advisory Committee Member for Sight UK, Retina UK, Macular Society

AUTHORS

**Aleksandra Sedova, MD**

Department of Ophthalmology and Optometry

Medical University Vienna

Vienna, Austria

**Dorottya Hajdu, MD**

Department of Ophthalmology and Optometry

Medical University Vienna

Vienna, Austria

**Felix Datlinger, MD**

Department of Ophthalmology and Optometry

Medical University Vienna

Vienna, Austria

**Irene Steiner, MSc**

Center for Medical Statistics

Informatics and Intelligent Systems

Section for Medical Statistics

Medical University Vienna

Vienna, Austria

**Martina Neschi, PhD**

Department of Ophthalmology and Optometry

Medical University Vienna

Vienna, Austria

**Julia Aschauer, MD**

Department of Ophthalmology and Optometry

Medical University Vienna

Vienna, Austria

**Bianca S Gerendas, MD, MSc, PhD**

Department of Ophthalmology and Optometry

Medical University Vienna

Vienna, Austria

**Ursula Schmidt-Erfurth, MD**

Department of Ophthalmology and Optometry

Medical University Vienna

Vienna, Austria

**Andreas Pollreisz, MD**

Department of Ophthalmology and Optometry

Medical University Vienna

Vienna, Austria

CME AUTHOR

Laurie Barclay, MD

Freelance writer and reviewer

Medscape, LLC

Disclosure: Laurie Barclay, MD, has disclosed no relevant financial relationships.
